# Assessing the effects of a novel biostimulant to enhance leafminer resistance and plant growth on common bean

**DOI:** 10.1038/s41598-021-98902-z

**Published:** 2021-10-08

**Authors:** Amr A. Mostafa, Soheir N. Abd El-Rahman, Said Shehata, Naglaa A. Abdallah, Hanaa S. Omar

**Affiliations:** 1 Department of Biochemistry, Faculty of Agriculture, Cairo University, Giza, Egypt; 2 Department of Crops Technology Research, Food Technology Research Institute, Agricultural Research Centre, Giza, Egypt; 3 Department of Horticulture, Faculty of Agriculture, Cairo University, Giza, Egypt; 4 Department of Genetics, Faculty of Agriculture, Cairo University, Giza, Egypt; 5 National Biotechnology Network for Experts, ASRT, Giza, Egypt; 6 GMO Laboratory, Cairo University Research Park, Giza, Egypt

**Keywords:** Biological techniques, Biotechnology, Molecular biology, Plant sciences

## Abstract

The leafminer *Liriomyza trifolii* is one of the major insects that affect *Phaseolus vulgaris* production worldwide. Novel and safe biobased stimulator compound (BSTC) with micronutrient-amino acid chelated compounds was developed from natural compounds and was used for foliar spray of *P. vulgaris*. Treated plants showed significantly increased in quality and productivity as well as significant reduction in leafminer infestation by close the tunnel end resulting in larvae suffocation and death. BSTC contains chemical composition that has important function in inducing immunity and resistance against insects, enhance plant growth and production. Also, HPLC showed that the assembled BSTC is rich in nucleobases than yeast extract (> 56 fold). Aminochelation zinc enhanced the rate of absorption of nutrient compounds and could participate in safe biofortification strategy. The expression of plant defense related genes under BSTC treatment revealed strong correlations between the transcription rates of defense related genes. Based on binding energies and interacting residues of six vital insect proteins, the best-docked complexes was obtained with disodium 5′-inosinate, delphinidin 3-glucoside and hyperoside. Obtained findings indicate that the foliar application of BSTC can enhance plant growth and productivity, uptake of important elements, expression of defense related genes and inhibit insect essential genes.

## Introduction

Common bean (*Phaseolus vulgaris* L.) is one of the most commonly used grain legume for the direct human consumption^1^. The majority of bean production is highly affected by climate changes and biotic stresses. There are more than 20 species of *Liriomyza* have been reported as being economically important. The leafminer, *Liriomyza trifolii* is invasive pest that cause great losses in several crops ^2,3^. The larvae feed and damage the foliage, while the female adults puncture plant tissue during oviposition, both stages reduce photosynthesis, cause defoliation and result in yield loss, while damage crop plants have a role in leaf damage as larvae primarily mine the palisade mesophyll, feed and damage the foliage^3,4^. They are difficult to control as they are protected inside the mines, although synthetic insecticides are the main strategy in the management of insect pests, these chemical substances increase insect resistance over successive generations, negative effects on non-target organisms and are threat to human health. Researchers from many countries have attempted to use biological control to manage these pests such as *Bacillus thrungiensis*^2^. However, there are no effective and safe insecticides against adults, and few effective larvicides. In addition, heavy use of chemical fertilizers and pesticides has harmful effects on beneficial soil microorganisms^5^. In that respect, safe and sustainable natural bioactive products are requested for insect control, as an alternative to harmful pesticides.

Recently, a great attention has been paid on developing natural and safety substances to improve plant growth, yield quality and quality. Foliar feeding is more environmentally friendly; efficient rate of absorption and nutrients can be directly uptake to plant tissues during critical stages of plant growth and development in comparison to soil fertilization. Natural amino acid chelation enhanced bioavailability of nutrients, enhance uptake, absorption and translocation of nutrients. Aminochelates are safe, enhance uptake, absorption and translocation of nutrients and can be used both for soil and foliar applications^6^. Previous studies showed that including aminochelate in nutrient solution could result in plant growth, damage recovery, higher chlorophyll concentrations and delay of leaf senescence^6–9^. Application of chelating agents increased plant growth and biomass in stressed plants^10^. Citric acid is an excellent chelating agent, binding metals in yeast hydrolysate by making them soluble. Due to its small size molecule, it can easily penetrate into the roots of plants and can be adsorbed fast^10^.

The systemic acquired resistance participate in the expression of plant resistance to pathogens and insects has two major pathways; one involves salicylic acid (SA), and another jasmonic acid (JA)^11–14^. Studies showed that JA and SA are vital hormones that promote plant defenses and boost immune responses against pathogen and insect attacks^15–17^. JA regulates plant’s defenses against herbivores, while JA-mediated pathway regulates defensive gene expression in plants^18,19^. In addition, Antioxidants and plant growth promoting compounds such as nitrogen components, salicylic acid (SA), ascorbic acid, etc. have been used as stimulators for increasing the yield and quality of crops and fruits^20,21^.

Many factors can affect the structure and biochemical functions of protein in vivo, leading to changes in biologic activity. In general, the function of defense proteins associated pathology and insect infestation are broadly based on binding with different ligands^22^. Molecular docking is extensively used in predicting the expected binding patterns of natural compounds as well as the important interacting residues between two molecules to understand the possible biological activity of the title molecule. For developing new inhibitors for any target protein, first structure-based screening and molecular dynamics of compounds followed by docking on the active binding site is used^23^.

In general, there are no previous studies on the effect of the safe bioactive molecules for insect control. Therefore, the overall aim of the present work was designed to develop and disclose the influence of formulated bioactive stimulator with aminochelate activity developed from natural important chemical composition on leaf minor resistance and growth quality of two cultivars of common beans. To address this aim, we investigated the presence of important chemical compounds using HPLC and GC–MS in the developed bioactive stimulator and explored their influence on gene expression of plant defense related genes, and listed the most binding molecules to vital insect proteins by molecular docking approach to understand the biological activities involved in gene inactivation.

## Results

### Chemical composition of the stimulator compound

The GC–MS data (Figure S1) shows the presence of bioactive constituents and indicate that BSTC contains potential agents that have essential roles in stimulating immunity, defense signaling and enhance resistance against many pathogens and insects such as Pyridine 1,2,3,6 tetrahydro-1,2-dimethyl-(CAS), Adipic acid, 2-decyl ethyl ester, Squalene, L- Glutamic acid 5-ethyl ester and Oleic acid. While L- Glutamic acid 5-ethyl ester and citric acid enhances plant Growth, tissue repair and photosynthesis. Also, cis-9-Hexadecenoic acid, dodecanoic acid, 3-hydroxy have essential role in cell signaling, membrane stabilizer and energy source.

Analytical analysis of the five nucleobases (adenine, thymine, uracil, guanine and cytosine) in nucleic acids detected using HPLC–DAD, showed that formulated BSTC contains high concentration of nucleobases with 56 fold of the yeast extract as the main source of nucleic acids (Figure S2).

### Effects of BSTC on plant growth

**Figure 1:**
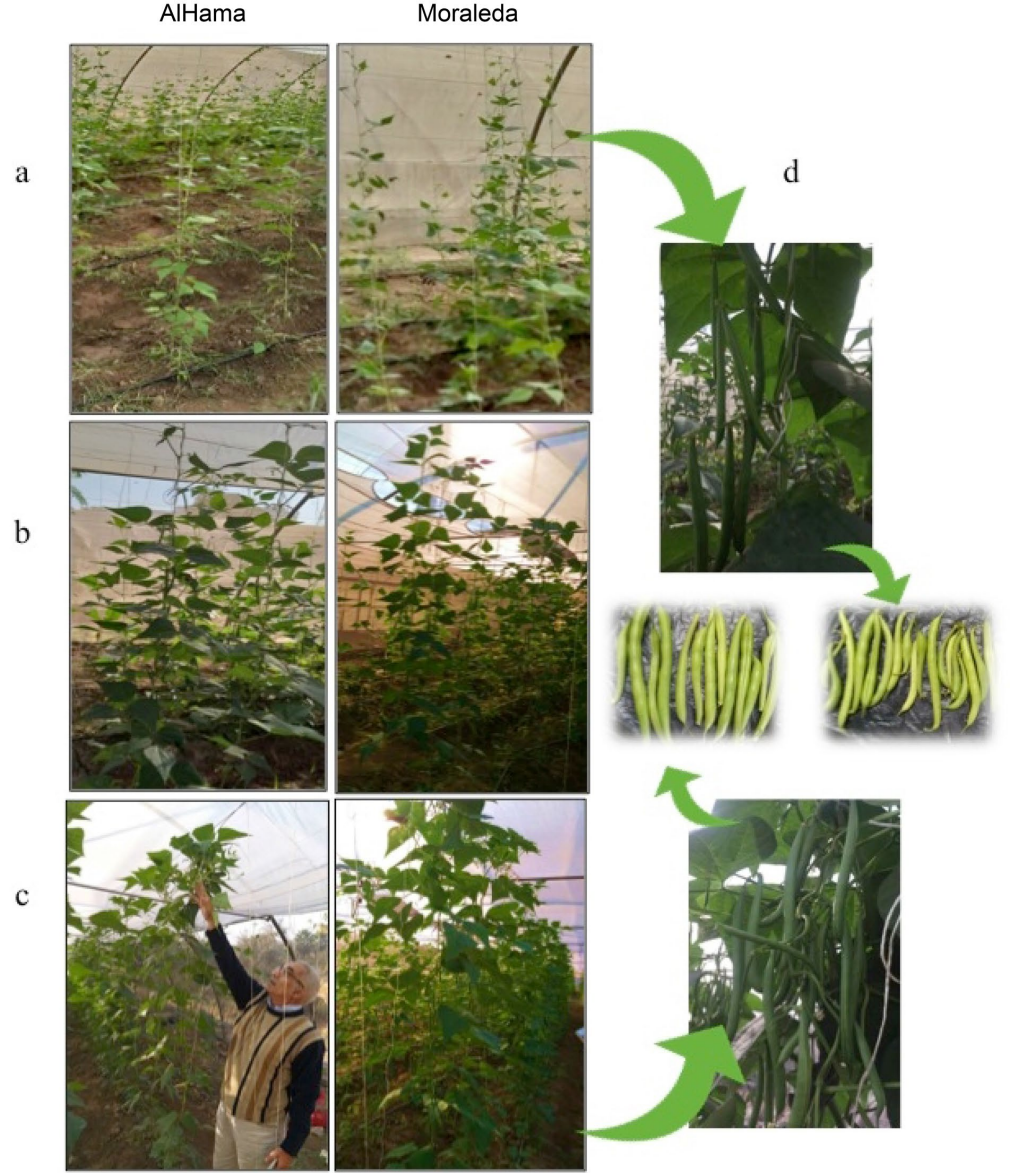
Vegetative and reproduction characteristics of foliar fertilized plants. AlHama and Moraleda plants (**a**) sprayed with water as negative control, (**b**) sprayed with yeast extract as positive control and (**c**) sprayed with growth promoting compounds. (**d**) Control plants gave thing and low yield pods, BSTC treated plants gave higher number and thicker.

The foliar application with BSTC on the two *P. vulgaris* cvs (AlHama and Moraleda) during the two consecutive seasons recorded significantly increase in several morphological characters of vegetative growth and yield, compared to the yeast extract application (C2) used positive control and the water sprayed cvs (C1) as negative control (Fig. 1). Significant differences were observed in growth parameters after BSTC treatments, especially plant height and yield, of both AlHama and Moraleda cvs during 2019 and 2020 seasons are presented in Table 1. Nevertheless, BSTC treated AlHama cv recorded maximum increases in vegetative growth. Compared to the negative control (C1), total yield increased 150% and 96%, in addition to significant increases in plant height increase and number of pods/plants for AlHama and Moraleda, respectively. While comparing to C2 (+ ve control sprayed with YE), total yield increased 70% and 11% for AlHama and Moraleda, respectively (Table S2). The differences between *P. vulgaris* cvs in vegetative growth parameters could be attributed to their variations in the genetic habitation and responses to the environmental conditions. In the case of C2 treatment, although it increase the growth parameters for the two cultivars but the increases were not significant compared to the control. Results revealed that the enhancement effect of the BSTC could be attributed to the satisfactory impact of them on the metabolism and biological activity and their unexpected effect on photosynthetic pigments and the enzyme activity, which could encourage the vegetative growth of *P. vulgaris*.

**Table 1:** Effect of the application of BSTC on the growth parameters of *P. vulgaris*, AlHama and Moraleda cvs, during 2019 and 2020 cultivating seasons.

Item	1st season									2nd season								
	Total yield (g)	Yield/Plant (g)	No.of pods	Pod weight (g)	Plant height (cm)	Leaves no	TSS	Thickness	Chlo	Total yield (g)	Yield/Plant (g)	No.of pods	PodWeight (g)	Plant height (cm)	Leaves no	TSS	Thickness	Chlo
**AlHama**																		
C1	290.63b ± 85.25	124.21c ± 7.53	36.33b ± 9.28	3.52b ± 0.25	157.67b ± 17.57	17.33b ± 1.45	3.80b ± 0.12	1.10a ± 0.06	45.40c ± 0.12	311.03b ± 42.18	129.00b ± 17.58	39.00c ± 1.15	3.63b ± 0.18	162.00b ± 14.53	18.00b ± 1.15	3.70b ± 0.15	1.10a ± 0.12	45.60c ± 0.12
C2	413.97b ± 49.49	174.33b ± 2.96	52.00ab ± 5.29	4.01b ± 0.12	189.67b ± 7.86	20.00b ± 1.00	4.13b ± 0.09	1.07a ± 0.12	47.20b ± 0.06	453.37b ± 33.86	189.00ab ± 26.63	57.00b ± 4.36	4.14b ± 0.26	195.33b ± 4.33	21.00b ± 1.53	4.20b ± 0.17	1.10a ± 0.15	47.80b ± 0.06
T	726.80a ± 66.65	242.26a ± 22.22	72.67a ± 8.11	4.90a ± 0.27	241.67a ± 7.26	25.33a ± 1.76	5.10a ± 0.15	1.30a ± 0.06	52.30a ± 0.06	754.87a ± 49.55	253.33a ± 17.70	77.00a ± 5.20	5.07a ± 0.14	251.00a ± 7.94	27.00a ± 1.15	5.00a ± 0.21	1.20a ± 0.06	52.40a ± 0.12
LSD	237.74	47.24	26.80	0.77	41.107	4.98	0.42	0.29	0.28	146.55	72.91	13.75	0.69	34.19	4.47	0.62	0.40	0.35
**Moraleda**																		
C1	387.33a ± 24.43	193.67a ± 12.22	26.00a ± 13.05	3.35b ± 0.19	219.00a ± 19.86	13.33b ± 1.67	3.83b ± 0.03	0.80b ± 0.12	44.30c ± 0.12	402.43b ± 14.77	201.57a ± 9.35	25.00b ± 5.57	3.37b ± 0.18	227.67b ± 12.47	13.00b ± 0.58	3.80b ± 0.12	0.90b ± 0.12	44.20c ± 0.23
C2	536.60a ± 22.69	208.20a ± 14.08	28.00a ± 1.73	3.93b ± 0.22	226.67a ± 18.56	16.00b ± 1.00	4.40a ± 0.15	1.00b ± 0.06	46.20b ± 0.12	564.33a ± 36.73	222.50a ± 7.23	27.00b ± 2.08	3.93ab ± 0.19	240.67b ± 12.47	16.00b ± 1.15	4.20ab ± 0.32	1.00b ± 0.06	46.60b ± 0.44
T	586.10a ± 156.79	222.36a ± 52.26	32.00a ± 9.02	4.71a ± 0.25	262.00a ± 18.56	22.00a ± 1.15	4.80a ± 0.15	1.40a ± 0.06	49.70a ± 0.06	631.67a ± 20.88	234.63a ± 30.04	31.00a ± 1.53	4.61a ± 0.28	282.67a ± 10.14	23.00a ± 1.53	4.70a ± 0.21	1.30a ± 0.06	50.00a ± 0.12
LSD	320.25	110.86	31.88	0.76	65.752	4.52	0.44	0.28	0.35	89.42	64.50	12.26	0.76	40.635	4.00	0.80	0.28	1.01

Averages with different letters have a significant difference at the 0.05 level.

C1 water sprayed plants as negative control.

C2 sprayed plants with yeast extract as positive control.

TSS, total soluble solids; Chlo, chlorophyll contents.

### Evaluation of foliar activators on Liriomza trifolii infestation

**Figure 2:**
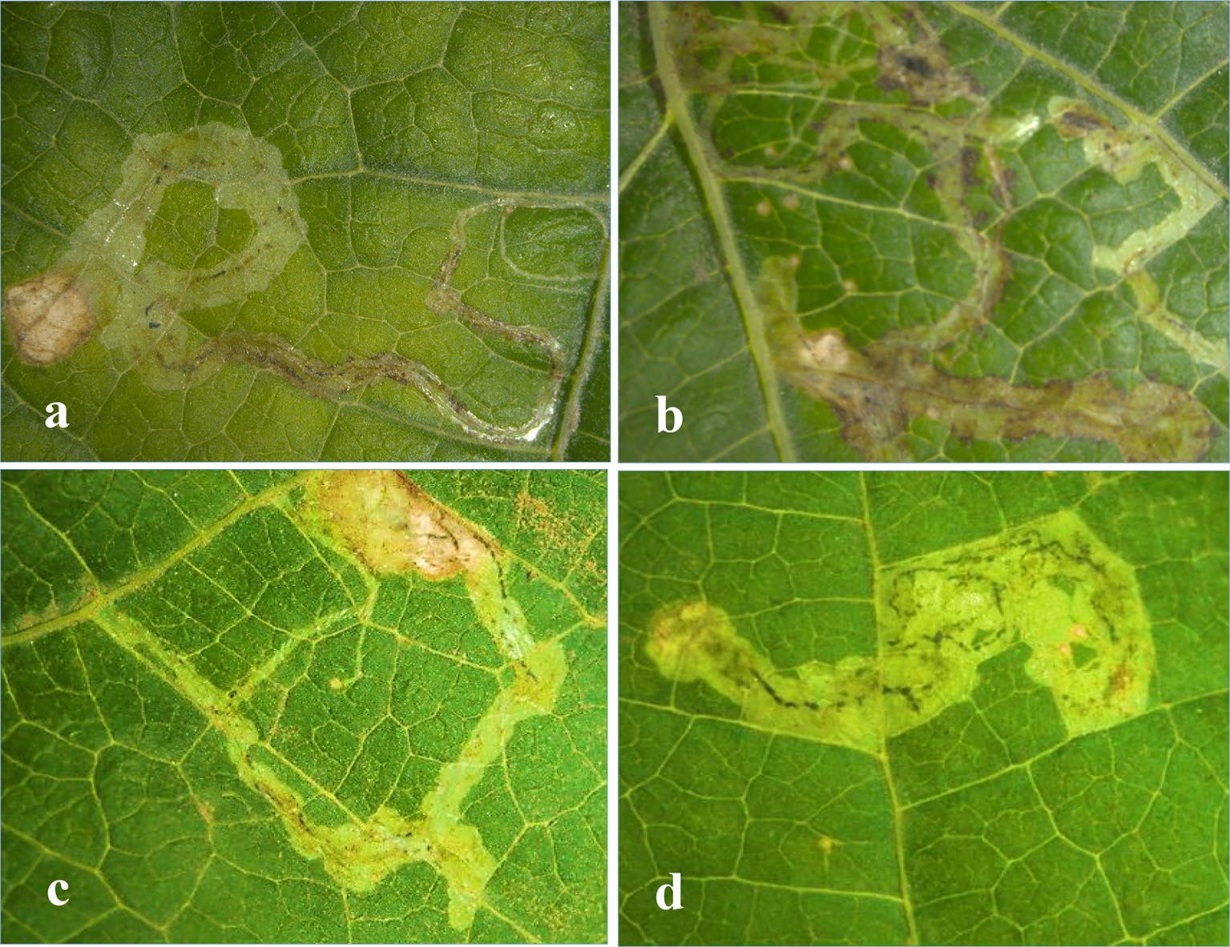
Damage caused by the larvae infestation of the *L. trifolii* in *P. vulgaris* cvs showing the effects of treatment with BSTC on resistance. Symptoms of *L. trifolii* on the leaves of *P. vulgaris* (**a**) AlHama control, (**b**) AlHama treated with BSTC showing dead larva, (**c**) Moraleda control and (**d**) Moraleda treated with BSTC with dead larva.

During the two cultivation seasons of the *P. vulgaris* cvs, the BSTC treated plants revealed significant decrease in the number of mines (> 62%) and mine length (60%) in the case of AlHama cv compared to the control, while the C2 treatment caused only 25% and < 4% decrease for the number of mines and mine length, respectively (Table 2). Moraleda cv, treated with BSTC caused less effect than AlHama (< 50% miner number, < 56% mine length), while C2 treated plants did not differ much than the C2 treated AlHama plants. Negative control as well as the C2 treated plants clearly showed that the ends of population tunnels are opened, the number of the mine/ leave were 6 and 8, respectively, the length of the mine was > 2 cm and the insects were able to complete the life cycle and reached the adult stage. On the other hand, BSTC treated plants caused decrease in length of mine (~ 1 cm), the number of mines/leaf (2–4) and the ends of population champers were closed that cause blocking the breathing holes of insects causing suffocation and death. BSTC treated plants showed dead larvae inside the tunnel (Fig. 2).

**Table 2:** Scoring infestation reduction form *L. trifolii* on foliar-sprayed *P. vulgaris* plants. Averages with no common letters have a significant difference at the 0.05 level.

Items	1st season 2019 Infestation rate (%) recorded weekly					2nd season 2020 Infestation rate (%) recorded weekly				
	1st	2nd	3rd	4th	5th	1st	2nd	3rd	4th	5th
**AlHama**										
C1	46.67 ± 13.3a	80.00 ± 3.8a	46.67 ± 6.6a	51.11 ± 8. 9a	35.55 ± 12.4a	51.11 ± 5.9a	55. 56 ± 11.8a	48. 89 ± 9.7a	40.00 ± 10.1b	40.00 ± 10.2b
C2	48.89 ± 9.6a	44.44 ± 5.8b	40.00 ± 3.8a	66.67 ± 3.8a	28. 89 ± 8.9a	37. 78 ± 4.4a	42. 22 ± 8.0a	35. 56 ± 5.9a	26. 67 ± 6.7b	26. 67 ± 6. 7b
BSTC	4.44 ± 2.2b	20.00 ± 10.18b	13.33 ± 7.7b	15.56 ± 9.7b	11.11 ± 5.9a	4.44 ± 2.2b	17. 78 ± 8.9ab	17. 78 ± 5.9ab	17. 78 ± 5.9b	17. 78 ± 5.9b
F values	6.804	17.839	7.875	10.974	1.796	29.250	3.912	4.485	2.054	2.054
*P* values	0.029	0.003	0.021	0.010	0.245	0.001	0.082	0.064	0.209	0.209
Reduction	90%	75%	71%	70%	68%	92%	69%	64%	57%	57%

Treatments	1st season 2019 Infestation rate (%)					2nd season 2020 Infestation rate (%)				
	1st	2nd	3rd	4th	5th	1st	2nd	3rd	4th	5th
**Moraleda**										
Control	53.33 ± 3.8a	55.56 ± 9.6a	64.44 ± 5.9a	64.44 ± 5.9a	62.22 ± 2.2a	46.67 ± 13.3b	66.67 ± 6.7a	55.56 ± 8.9a	77.78 ± 4.4b	46. 67 ± 6.7a
YE	37. 78 ± 5.9a	51.11 ± 5.8a	42.22 ± 9.7a	51.11 ± 5.9b	37.78 ± 11.8a	31.11 ± 5.8b	40.00 ± 3.8b	66.67 ± 7.7a	53.33 ± 11.5b	33.33 ± 7.7a
BSTC	11.11 ± 5.9b	15.56 ± 5.8b	26.67 ± 7.8ab	40.00 ± 6.7c	17.78 ± 9.6ab	20.00 ± 3.8b	28.89 ± 8.0c	26.67 ± 13.9ab	37.78 ± + 15.5b	24.44 ± 4.4a
F values	16.294	8.848	5.763	3.957	6.271	2.370	9.160	3.866	3.088	3.040
*P* values	0.004	0.016	0.040	0.080	0.034	0.174	0.015	0.083	0.120	0.123
Reduction	79%	71%	58%	38%	25%	57%	56%	51%	51%	48%

In addition, the reduction rate (Table 3) of the leafminer infesting AlHama cv were significantly decreased during five weeks after the application of the BSTC treatments compared to the controls (C1 and C2) from 90 to 68% in the 1st season and from 92 to 57% in the 2nd season. In the case of Moraleda, reduction rate decreased from 79 to 25% in the 1st season and from 57 to 48% in the 2nd season. Reduction rates indicate the stability, effectiveness and persistence of the BSTC compound on the *L. trifolii* larvae insect for five weeks in 2019 and 2020 seasons. The results revealed that BSTC treatment increased the plant resistance to insect, but AlHama was more resistant than Moraleda. The mechanisms by which growth promoter compounds found in BSTC mediate disease resistance remained unexplored and elusive, yet its exact function has remained unclear. Therefore, gene expression of defense related genes were studied.

**Table 3:** Average numbers of mines and tunnel length per leave produced by larvae of *L. trifolii* in common bean sprayed with BSTC, YE and water during cultivating seasons 2019 & 2020.

Items	AlHama				Moraleda			
	1st season		2nd season		1st season		2nd season	
	Mines/leave	Main mine length (cm)	Mines/leave	Main mine length (cm)	Mines/leave	Main mine length (cm)	Mines/leave	Main mine length (cm)
C1	8.0 ± 1.14a	2.2 ± 0.15a	8.0 ± 0.8a	2.02 ± 0.29a	8.0 ± 2.1a	2.16 ± 0.26a	8.0 ± 0.89a	2.28 ± 0.17a
C2	6.0 ± 0.89a	2.14 ± 0.3a	6.0 ± 0.7b	1.88 ± 0.16a	6.0 ± 0.84a	1.98 ± 0.12a	6.4 ± 0.67a	1.92 ± 0.08b
BSTC	2.0 ± 0.31b	0.9 ± 0.2b	3.0 ± 0.0c	0.8 ± 0.3b	4.0 ± 1.0a	1.04 ± 0.07b	4.6 ± 0.92b	1.06 ± 0.06c
T-Value	12.727	10.566	15.833	6.498	1.967	12.248	4.094	29.919
*P*-Value	0.001	0.002	0.000	0.012	0.182	0.001	0.044	0.000

C1 water sprayed plants as negative control.

C2 sprayed plants with yeast extract as positive control.

### Expression of biotic stress-related genes in P. vulgaris during L. trifolii attacks

**Figure 3:**
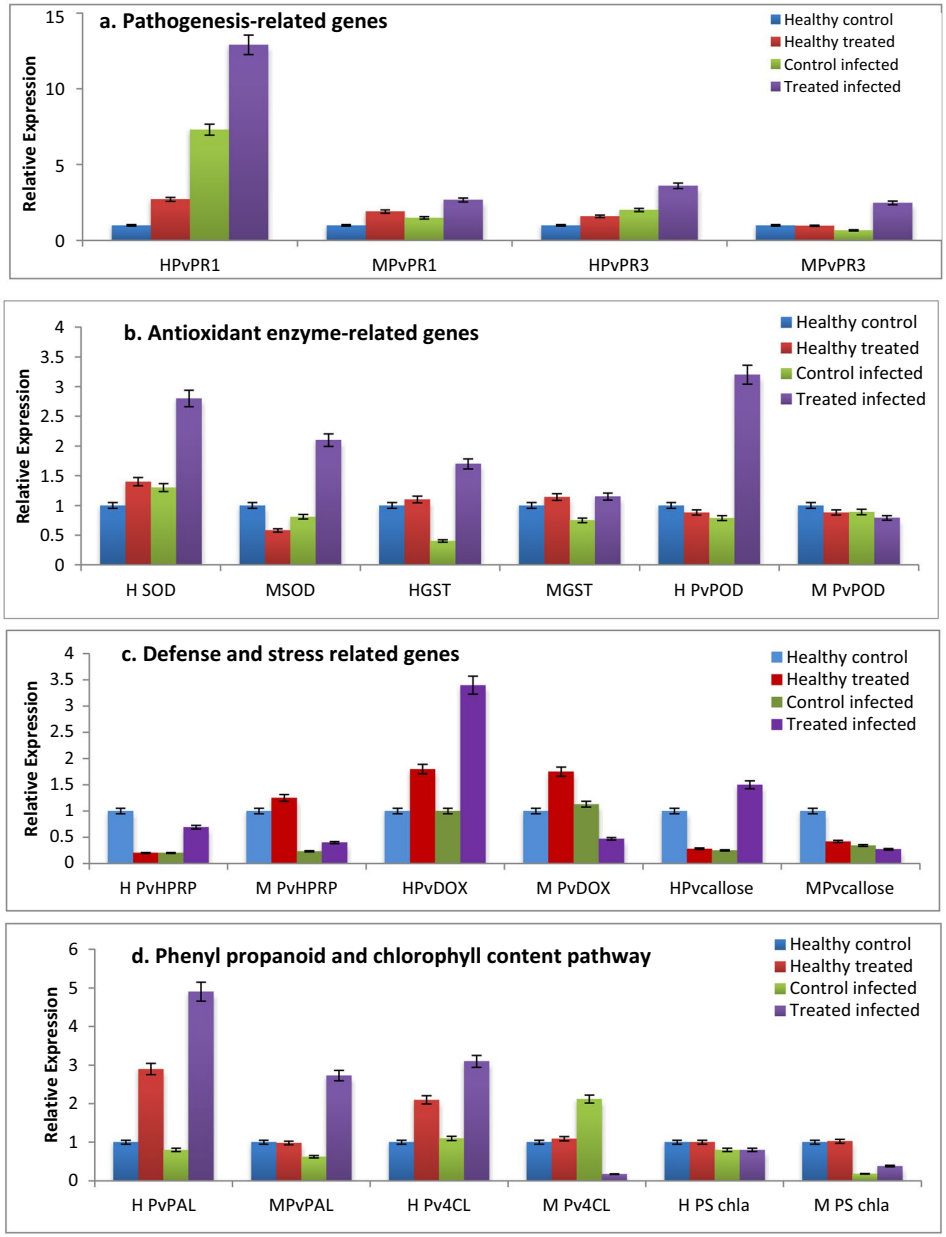
Differential expression of defense related genes in *P. vulgaris* AlHama (H) and Moraleda (M) cvs 2 days post infestation with *L. trifolii*. (**a**) Pathogenesis related genes (PvPR1 and PvPR3), (**b**) antioxidant enzyme-related genes (PVDOX , SOD and GST), (**c**) defense and stress related genes (PVOD, PvHPRP and Pvcallose) (**d**) phenyl propanoid pathway (PVPAL and PV4CL) and light-harvesting type-I (Lhcll-I ).

Plants respond to pathogen or insect attacks by activating the synthesis of a diverse number of defense proteins. Corroborating this, comparative analysis of disease related genes of the BSTC treated common bean with C1 and C2 plants under leafminer infestation were carried out as an attempt to understand the indirect effects of BSTC compounds insect infestation. The expression profile of 11 functional genes belonging to the four important groups of the identified genes related to biotic stress response were evaluated in order to clarify the plant response during the interaction with the leafminer *L. trifolii*: pathogenesis related (PvPR1 & PvPR3), antioxidant enzyme-related genes (PvPOD SOD & GST), defense and stress related (PvHPRP, PvDOX & Pvcallose), Phenyl propanoid pathway (PvPAL & Pv4CL) groups in addition to the light-harvesting chlorophyll type-I (Lhcll-I) were evaluated using RT-PCR two days post infestation with *L. trifolii* (Fig. 3). Significant fold differences for the expression of selected genes in the treated plants with BSTC after leafminer infestation were observed. The expression levels for pathogenesis related genes; PvPR1 (Pathogenesis-related) and PVPR3 (Chitinase class I) were increased two days post infestation with the leafminer in both AlHama and Moraleda cvs and were significantly increased with plants treated with BSTC. The expression of PvPR1 increased 1.5 fold following leafminer infestation in BSTC treated compared to the untreated plants. The results revealed that the transcription level of the SA-inducible genes PR1 and PR3 plays an important role in the defense-response in plants.

For the antioxidant enzyme related genes; PvPOD (peroxidase), SOD (Superoxide dismutase) and GSTT (glutathione S-transferase), leafminer infestation decreased their expression, but BSTC treatment caused significantly up-regulation of those enzymes indicating the capability of the SOD in the activation of antioxidant defense systems in plants. That indicates that BSTC may have important chemical compound that result in stimulating the PvGST and PvSOD gene expression.

Additionally, the transcript levels of the defense and stress related genes; PvDOX (α-dioxygenase), Pvcallose (Callose synthase) showed high transcript levels during the early stages of the interaction *L. trifolii*–*P. vulgaris* interaction, while PvHPRP (Hypersensitive-induced) showed decrease expression level in the later stages of infestation. In the case of AlHama cv, the down-regulation of the defense and stress related genes might explain the lower level of resistance and growth compared to AlHama. Those genes known are largely responsible for JA induction genes indicating that JA has important roles in the plant response to leafminer.

Moreover, the genes involved in the phenyl propanoid pathway; Pv4CL and PvPAL (phenylalanine ammonialyase), were upregulated in 2 days post interaction with *L. trifolii*. Although the expression of the Pv4CL (4-coumarate CoA-ligase) was significantly down regulated. However, the gene Lhcll-I, involved chlorophyll content pathway, showed down-regulation due to the *L. trifolii* infestation even after BSTC treatment. That indicates that leafminer infestation affects the chlorophyll content as well as plant growth. The expression of the Lhcll-I gene showed down regulation due to leafminer infection, although BSTC treated plants revealed a slide increase in its expression.

### Effect of BSTC phytochemicals on L. trifolii vital proteins using docking analysis

Molecular docking is extensively used to model the interaction between a small molecule and a protein at the atomic level to elucidate the binding patterns of small molecules within the receptor active cite of the target protein.

**Figure 4:**
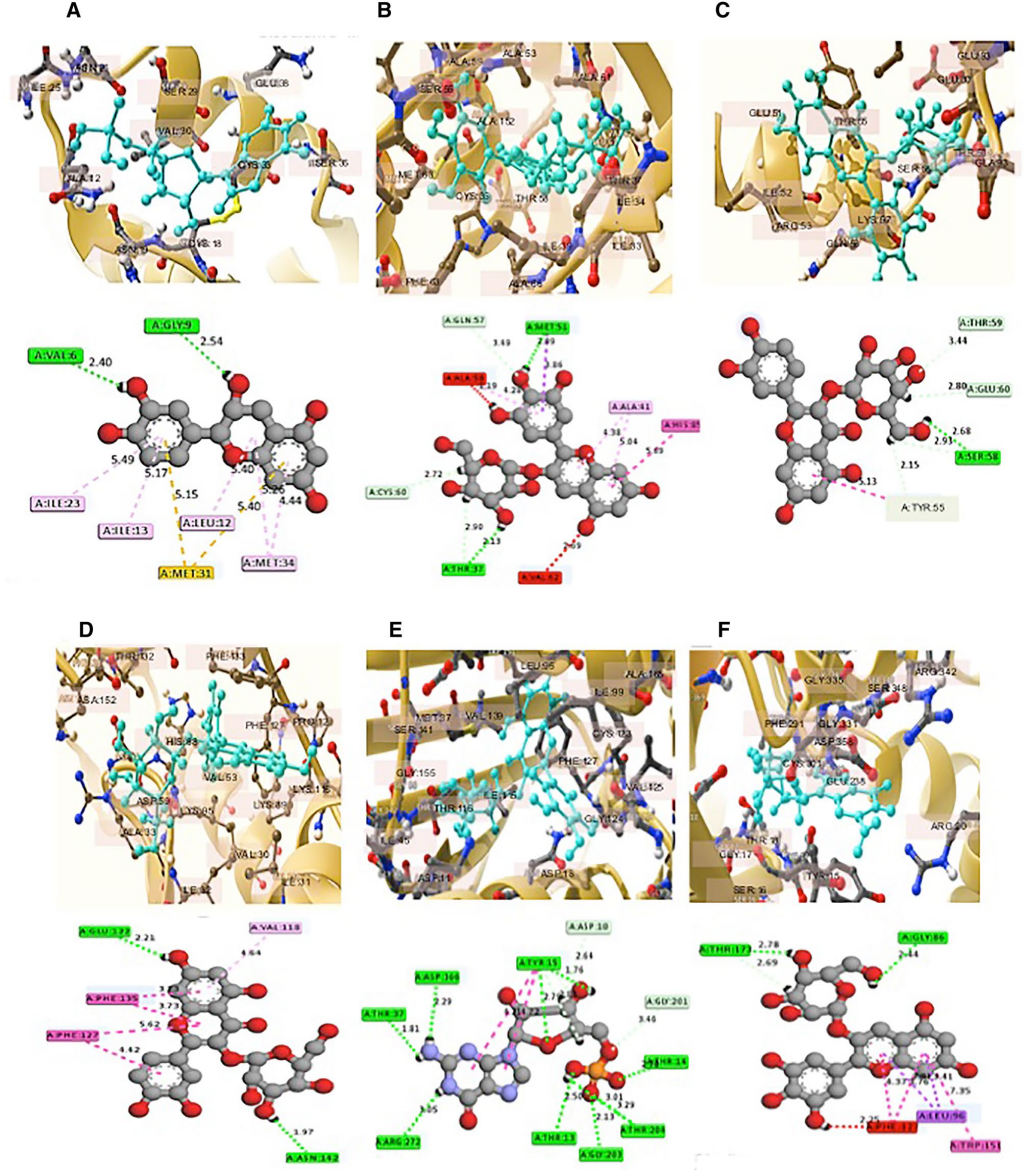
The 2D and 3D diagrams of acetylcolenestraea with Disodium 5'-inosinate (**A**), elongation factors with delphinine 3-glucoside (**B**), histone subunit3 with hyperoside (**C**), arginine kinase with hyperoside (**D**), HSP70 with disodium 5-guanylate (**E**), HSP90 with Delphinine 3-glucoside (**F**) virulence insect proteins. Molecular docking was built uisng SAMSON software 2020.

In the current study, we screened various natural compounds presented in the BSCT with 6 vital proteins that have essential roles in pathway of life cycle of the insect: acetyl cholinesterase, elongation factor, histone subunit3, arginine kinase, HSP70 and HSP90. To assess their stability and reliability, a range of methods were used to estimate the efficiency of the predictive model acetyl cholinesterase, elongation factor, histone subunit3, arginine kinase, HSP70 and HSP90 protein model. PROCHECK analysis, which quantifies the residues in the Ramachandran plot, was used for eventually validation. The ERRAT tool, which defines the protein’s overall quality factor, was used to study statistics on no-bonded interactions among different types of atoms. The molecular interactions of protein–ligand were docked by SAMSON 2020 software through predicting the potential affinity, molecular structure, geometry optimization of structure, vibration frequencies of coordinates of atoms, bond length and bond angle. The best scoring poses out of 100 poses were used to calculate the binding affinity of the ligands. Shortlist of screening exhibit best binding ligands to the selected proteins is presented in Table S3. Ligand with the highest binding score with each protein was identified. The grid was configured with the following parameters: size X, Y, Z, structural properties of each molecule and calculated binding affinities to the six model proteins for *L. trifolii* insect. Based on docking results, we shortlisted of three compounds, disodium 5'-inosinate binds exhibit best binding with acetyl cholinesterase and HSP90 exhibited, delphinidin 3-glucoside best binds with elongation factor and HSP90, while hyperoside best binds histone subunit 3, arginine kinase and cytochrome with binding score ranged between − 10.2 to − 5.0 (Table S3). The best-docked compounds were evaluated for their 2D and 3D structures and the important interacting residues obtained during molecular docking (Fig. 4). The predicted docking binding energy point to the possible of biological activity of the title molecule on inhibiting the protein function and may participate in insect mortality.

### Discussion

Foliar application of soluble biochemical represents an additional source for delivering essential nutrients to plants biofortification^24^. Leaves can uptake the nutrients either through penetrating the cuticle or through the stomata^24^. A safe and environmental friendly compounds is required for cropping production systems^25^. In this research, we highlighted the effects of applying a new, inexpensive and easy to prepare biobased stimulator compound on enhancing growth development, yield and leafminer resistance of common bean under normal agronomical practices. BSTC is prepared from compound obtained from yeast autolysate as a rich source of high quality protein, essential amino acids, essential minerals and vitamins that enhance cell division and enlargement^26^. The prepared suspension is supplemented with citric acid and salicylic acid that enhance plant growth^27^ and ascorbic acid which has an essential role in plant cell division, cell expansion, growth and development.

### Effects of chemical composition and micronutrient chelated compounds

**Figure 5:**
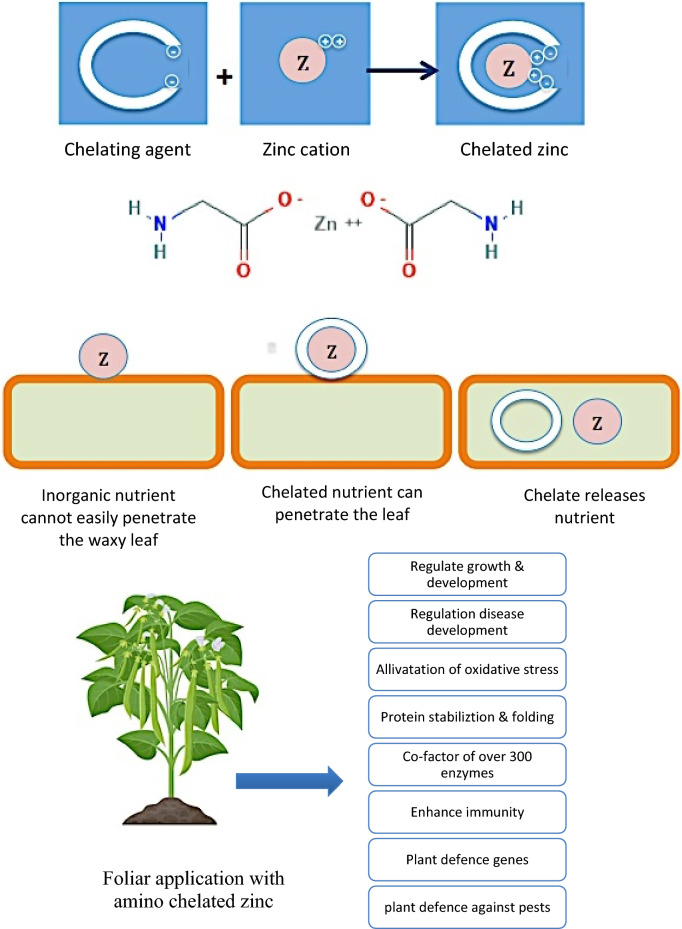
Diagram for chelation mechanism of zinc. Waxy leaves repels inorganic substances nutrient to penetrate into leaf. The organic coating around the chelated nutrient allows it to penetrate through the wax. Once entered the leaf, the chelate releases the nutrient and the plant can use it. Chelated zinc has many positive effects on plant growth and defense.

Micronutrient metals such as iron and zinc are essential nutrient elements required for healthy plant growth^6^. Zinc is an essential micronutrient that works as a co-factor of over 300 enzymes in plants and it plays important roles in protein synthesis, regulation of growth and development disease resistance^28^. Therefore, the presence of zinc chloride in the formulated BSTC allow zinc chelating by amino acids to improve foliar uptake in plants (Fig. 5). The aminochelatos are used to overcome iron and zinc deficiencies correction in plants^27^ and evolve plant defenses against pests and pathogens^29^. Several researches reported the effect of macronutrient fertilization and damage by insect herbivory^30,31^. From another perspective, high concentrations of amino acids presented in the formulated BSTC are considered as precursors for protein synthesis, indicating their importance for stimulation of cell growth and stimulate defense against pests and pathogens^27^.

The HPLC–DAD used for nucleobases analysis presented in this work has the potential to trace the nitrogen and carbon derived from nucleic acids. HPLC results revealed that the formulated BSTC has high concentration of nucleobases, one of the key elements that enhance growth and insect resistance in treated plants as they increase the absorption of the nutrient compounds. This is the first time using nucleobase as source of nutrient in plants. The presences of high concentration of nucleobases have an essential role in growth performance, and development of immunity when combined with yeast extract. Nucleotides and nucleosides are conditionally essential nutrients in tissues requiring rapid cell replication^32^ and development of immunity^32^. They are safely added to infant formula and animal feed ingredients^33,34^ to regulate growth performance and reduce the susceptibility to various stressors and diseases, which was correlated with enhanced growth.

The chemical composition of BSTC revealed from GC–MS showed that it contains important chemicals that may have essential role induce immunity and resistance against many pathogens and insects, enhance plant growth, and defense signaling that may lead to enhancing plant growth and insect resistance in a direct or indirect manner. Previous documents showed that adipic acid ethyl ester and pyridine 1,2,3,6 tetrahydro-1, 2-dimethyl both induce immunity and induces resistance against many pathogens and insects^33–35^; oleic acid that induces defense signaling, enhanced salicylic acid (SA) and exhibit enhanced resistance to multiple pathogens in plant^35^ and the squalene that mediates steroid synthesis and stimulates the immune system^36^. Another chemicals found in BSTC could be involved in stimulating of gene expression of essential genes in the plant cells.

### Effects on plant growth and yield

The foliar application with BSTC on the two *P. vulgaris* cvs (AlHama and Moraleda) during the two consecutive seasons recorded significantly increase in several morphological characters of vegetative growth and yield, compared to the C1 and C2 cvs. The foliar application with BSTC on the two *P. vulgaris* cvs (AlHama and Moraleda) during the two consecutive seasons recorded significantly increase in several morphological characters of vegetative growth and yield, compared to the C1 and C2 cvs. Nevertheless, BSTC treated AlHama cv recorded maximum increases in vegetative growth, as the yield increased more than double increased and plant height increased 50% compared to the Moraleda cv, that may be due to change in the genetic background between the two cvs which may act different with the foliar fertilizers. There is evidence that nutrient supply from the YE increase growth parameters in date palm^37^, soybean^38^, grape^20^, sweet potato^39^, pea^40^ and snap bean^41^. Several bioactive compounds were used as plant growth regulators to enhance crop quality such as salicylic acid, nitric oxide, jasmonic acid, ascorbic acid and citric acid were used to improve the immunity, yield and quality of various crops^42^.

### Effects on leafminer resistance

Apart from growth improvements, a new phenomenon was observed on the BSTC treated plants, the leafminer were not able to continue their life cycle causing the death of the larvae. The leafminer *L. trifolii* insect is an economically important insect that causes great losses in several crops^2,3^. Usually, synthetic insecticides and biological control are used to control leafminers infestation^2,5^. We recorded significant reduction in infestation after foliar spraying with BSTC with both *P. vulgaris* cvs compared to the controls. Also, treating infected plants showed close at the end of the tunnel causing larvae suffocation and death. In general, differences in leafminer infestation between the two cvs during the two tested seasons were insignificant. Results indicate that BSTC may contain stimulator to increase plant resistance to leafminer. No previous report indicated the effect of any foliar applications with natural compounds on insect infestation.

In this investigation, we developed for the first time safe and easily prepared biobased stimulator compound to control leafminer infestation.

Nevertheless, the mechanisms by which growth promoter compounds found in BSTC mediate disease resistance remained unexplored and elusive, yet its exact function has remained unclear. Therefore, gene expression of defense related genes were studied. Adding ascorbic acid, citric acid and salicylic to the formulated BSTC has positive effects on stimulating the plant growth hormones JA and SA., which play important roles in the plant metabolism in response to biotic and abiotic stresses^15,43,44^. In general, JA-mediated signaling pathways are implicated in the regulation of insect defenses, while the SA pathway is associated with defense responses against pathogens^15^.

### Effects on expression of defense related genes

Plants respond to pathogen or insect attacks by activating the synthesis of a diverse number of defense proteins. Corroborating this, comparative analysis of disease related genes of the BSTC treated common bean with healthy plants under leafminer infestation were carried out. The expression of the pathogenesis-related genes encoding antibiotic protein; *PvPR1* and *PvPR3* increased upon insect attach, but the expression of *PvPR1* increased 1.5 fold following leafminer infestation in BSTC treated compared to the untreated plants. In accordance with these findings, the upregulation of *PvPR1* was reported during the incompatible interaction between the common bean and fungal infection^45^. While, *PR3* gene (chitinase) induces signaling molecules that may work as defensive mechanism elicitors^43,44^.

The antioxidant enzyme-related genes; *PvPOD*, *PvGST* and *PvSOD,* presented a progressive increase in expression following infectious development in BSTC treated plants. That indicates that BSTC has important chemical compound that stimulate the *PvGST* and *PvSOD* gene expression. Additionally, the high expression of the defense and stress related genes; *PvDOX*, *PvHPRP* and Pvcallose, may indicate their role in insect infestation, except for the PvHPRP expression that showed down regulation.

The expression responses of defense and stress related genes observed in the present study are in agreement with the results of previous investigations. During the interaction between *Rhizoctonia solani*–*P. vulgaris,* highly expressed of *PvDOX* was recorded to protect plant tissues by undergoing excessive necrosis associated with oxidative stress during pathogenesis^44,45^. In fact, oxidative stress improves the ROS scavenging capacity in plant by increasing the expression of related enzymatic activities, such as glutathione S-transferase^45^. Also, *PvHIPRP* expressed under hypersensitive responses, was induced at the early stages of infection in infected tissue of *P. vulgaris*^46^. Plants unable to undergo programmed cell death^47^ or incapable of generating HR^’^ are more resistant to such pathogens. On the other hand, callose induction is an effective barrier presented at early stages of pathogen invasion and is associated with incompatible responses^48^. While the genes involved in the phenylpropanoid pathway, the *PvPAL* and *Pv4CL*, upregulated during infestation in AlHama cv but not with *Pv4CL* gene in Moraleda cv. That may explain the differences in response in the two cvs upon insect infestation.

On the other hands, the expression level of the *Pv4CL* gene in *P. vulgaris* infected with fungi was recorded as transient expression, with the greatest upregulation in the early stages of infection^46^. In Arabidopsis, mutation analysis in the PAL pathway caused rapid production of SA associated with local cell death^49^. Certain phenylpropanoid compounds, such as *PvPAL* and *Pv4CL*, leads to the synthesis of lignin, and many defense molecules, which are crucial for plant defense against abiotic and biotic stress factors^50^.

The expression of the *Lhcll-I* gene showed down regulation due to leafminer infection, although BSTC treated plants revealed a slide increase in its expression. *Lhcll-I* encodes the chlorophyll a-b binding protein which work as receptor for light harvesting, it captures and delivers excitation energy to photosystems. Its expression is regulated by multiple environmental and developmental cues^51^.

Due to the overexpression of *PvPOD*, *PvDOX*, *PvPAL*, *Pv4CL* Pvcallose in BSTC treated AlHama we hypothesize that they are involved in defense mechanism against leafminer infestation.

### In silico studies for molecules inhibiting the protein function

Molecular docking revealed that the main ligands supplemented by BSTC for bind the selected insect proteins are delphinidin 3-glucoside which binds with the insect HSP90 and elongation factor with high score (− 10.2 and − 9.2 kcal/mol, respectively), followed by disodium-5′-guanylate that bind with HSP70 with -9 kcal/mol while the binding energy of hyperoside binds with arginine kinase and histone subunit3 by − 8.8 and − 7 kcal/mol, respectively. The obtained results indicate that those ligands may have an essential role in insect death. In general, molecular docking revealed that binding the BSTC ligands have high docking scores with the insect essential proteins and could have negative effects in insect survival^52,53^. From the obtained results, we concluded that controlling leafminer infestation with BSTC implementation has positive influence on plant growth and yield quality and quantity improvement. However, further research is needed for mode of action of the BSTC that cause insect death and whether the BSTC have positive effects on controlling other harmful insects.

## Conclusions

Inexpensive, safe and easily prepared biobased stimulator compound were formulated with higher nutrient values was developed to avoid the use of any toxic auxiliary substances. The foliar application of the formulated BSTC have four main effects; (1) enhance plant uptake of micronutrients, (2) enrich plant with nutrients and nucleobases, (3) activate plant defense resulted genes and (4) inhibit insect essential proteins. BSTC treatment allows leaves to uptake of micronutrient such as zinc through aminochelting process. Overall, the finding presented in this study indicate that foliar application of the BSTC enhanced the *P. vulgaris* growth through foliar fertilization, as well as resistance to *L. trifolii* plants through increasing the expression of defense related genes. We suggest that the harmful effects provoked by leafminer stress could be alleviated by the exogenous application of the developed plant biobased stimulator compounds (BSTC) in common bean plants. It is important to note that nucleobases are important to increase plant absorption of useful nutrients, enhance immunity, vegetative growth and plant resistance to leafminer. This is the first report in using nucleobases as main compounds of foliar fertilizers for enhancing growth and controlling leafminer in common bean. This approach is particularly interesting in the current context of the damage caused by leafminer, as it is prepared from natural compounds, safe and inexpensive. We proposed that the developed BSTC represents a safe and good opportunities for the development of commercial for plant protection and growth enhancement.

## Materials and methods

### Preparation of growth promoting compounds

Bioactive stimulator is prepared from chemical composition obtained from autolysate yeast. For preparation, 200 g/l of bakery yeast (*Saccharomyces cerevisiae*) in deionized water then incubated at room temperature for 12 h to allow autolysis process. The suspension was heated at 100 °C for 10 min followed by fast cooling on ice to prevent enzymes from further digestion and to obtain high amount of useful compounds and nucleobases. The lysate was centrifuged at 4 °C in order to remove unbroken cells, partially disrupted cells and cell debris. To prepare the BSTC, ascorbic acid (250 ppm/l), citric acid (900 ppm/l) and salicylic acid (100 ppm/l) were added to the suspension. After 12 h incubation at room temperature 1 mM phosphoric acid and zinc chloride were added to previous mixture. The detailed method of preparing the BSTC was submitted to the Egyptian patent office (No. 1232/2020). As a control, yeast extract (YE) was prepared as described by^53^.

### Chemical analysis

Chemical composition of the BSTC was analyzed by gas chromatography-mass spectrometry (GC–MS) using Thermo Scientific, Trace GC Ultra/ISQ Single Quadrupole MS, TG-5MS fused silica capillary column with electron ionization mass detector^5^. For separation and quantitative measure of the nucleobases in the BSTC suspension, high performance liquid chromatography using Agilent HPLC 1100 system equipped with an auto sampler, diode array detector (DAD) and ChemStation software^54^.

### Field experimental design

All experimental research complied with the Ministry of Agriculture and Land Reclamation bulletin with the most important technical recommendations. Two experiments were carried out under experimental net-house condition at the East-wing farm of the Faculty of Agriculture, Cairo University during the early Summer season (cultivated in mid of February and harvested in June) of 2019 and 2020 to study the effect of foliar fertilizers on vegetative growth, pod yield and quality as well as leafminer resistance for two common bean cvs (AlHama and Moraleda). Seeds were bought Belco, Egypt (www.belco.com), the original source of AlHama is Monsanto Holland BV, while Moraleda source is Seminis Company.

The experiment involved 18 plots each contain 150 plants; 9 plots were cultivated with AlHama, other 9 with Moraleda. The following foliar fertilization were used:

- Three plots out of the nine plots were foliar sprayed with the BSTC, three plots was spread with YE and three plots were sprayed with water and used as control.
- Spraying was carried out three times during the growth period of bean plants at 30, 40, 50 days from sowing.

Data were recorded 61 days after sowing (flowering stage), 50 plants were randomly marked from each plot for determining the Plant height, number of leaves/plant, total soluble solids (TSS) and total chlorophyll content in leaves was measured using SPAD units through monitoring of chlorophyll meter (SPAD- 501). After maturation, crop yield and its components were estimated such as weight and thickness of pods/ plant (g), number of pods/plant and total yield/plant (g). Green pods of each plot were harvested counted and weighted.

Additional experiment was carried at the experimental greenhouse with 80 plants cultivated pots (40 plants from each cv) for studying gene expression under leafminer infestation. Twenty pots from each cv were covered with plastic bag to prevent infection. Three weeks after sowing plants were sprayed and divided into four groups; (1) infected sprayed with water/covered, (2) infected spayed with BSTC/covered, (3) healthy sprayed with water and (4) healthy with BSTC. Leaf samples were collected twice; 2 days post spraying for estimating gene expression.

Data of treatment means were compared using least significant difference (LSD) method at 5% significance level. All statistical analyses were performed by analysis of variance technique using CoSTATE computer software. Duncan Multiple Range Test at 5% was used to test the significant differences between the means of the different treatments.

### *Liriomyza trifolii* infestation to *P. vulgaris*

During the two seasons cultivation, the two *P. vulgaris* cvs AlHama and Moraleda were infested with the leafminer *Liriomyza trifolii.* Infection were measure at the upper, middle and lower level leaves/plant to count the average number of mines per leaf, length of mine per leaf and infestation percentages for 5 weeks for sprayed plants with BSTC and the controls; YE (C2) and water (C1). For calculating the infestation reduction, infestation was recorded by counting mines and/or live larvae weekly for five times post foliar treatment (30 days from sowing).

### Quantitative real-time PCR (qRT-PCR)

Total RNA was extracted from leaf samples collected from the sprayed plants cultivated in contained greenhouse and artificially infected with *L. trifolii*, using the RNeasy® Plant Mini Kit (Qiagen, Hilden, Germany) according to the manufacturer instructions. A total of 1 to 2 μg of DNase I treated RNA was reverse transcribed using Prime-Script First Stand cDNA Synthesis Kit (thermo kit). cDNA was analyzed by quantitative real-time PCR using SYBR kit (Takara) in Thermal Cycler Bio-Rad Real-Time System II (- (Bio-Rad, California, USA).

Primers specific for biotic stress expressed related genes^46,55,56^ were designed using primer blast (https://www.ncbi.nlm.nih.gov/tools/primer-blast/) and primer3 (https://primer3.ut.ee/) software. Those selected genes covered the main groups of defense genes including; pathogenesis related genes (Chitinase *PvPR3* & Pathogenesis-related protein 1 PvPR1), antioxidant enzyme-related genes (Peroxidase PvPOD, superoxide dismutase SOD & Glutathione S-transferase PvGST), defense and stress related genes (Hypersensitive-induced response protein PvHPRP, α-dioxygenase PvDOX & Callose synthase-like protein Pvcallose), Phenyl propanoid pathway (phenylalanine ammonialyase PvPAL & 4-coumarate CoA-ligase *Pv4CL*) groups as well as the light-harvesting chlorophyll type-I (Chlorophyll a/b-binding protein Lhcll-I). Primers were used in RT-qPCR analysis to amplify fragments of ~ 80–200 bp in length (Table S1) using the Takara SYBR® Premix Ex *Taq*™ in 25 μl reactions containing 60 ng template cDNA and 5 μl of 1 μmol/l of each oligonucleotide, 12.5 μl SYBR premix ExTag. Amplifications were performed by initial denaturation at 95 °C for 120 s followed by 40 cycles at 95 °C for 15 s and at 60 °C for 30 s. Melting curves were analyzed for each data point to check the specificity of the PCR product. For different gene expression results, the delta-delta Ct was calculated. Average CT values were calculated from the triplicate experiment conducted for each gene as the CT value detected by subtracting the average CT value of genes from the CT value of actin gene. Actin1 was used as housekeeping genes to normalize cDNA concentrations.

### Statistical analysis

The statistical analysis was conducted through the Tukey’s test at the 0.05 level and data were analyzed using Two-way ANOVA computer program. The accepted level of significance (*P* ≤ 0.05) was considered. Means of measured traits were compared using L.S.D. at 0.05% level of probability.

### Molecular docking analysis

In the absence of a crystallographic structure for acetyl cholinesterase, elongation factor, histone subunit3, arginine kinase, HSP70 and HSP90 from L. trifolii, its three dimensional structure was obtained by homology modelling. The SWISSMODLE server (https://swissmodel.expasy.org/)^57^ was used to predict the 3D structure of the acetyl cholinesterase, elongation factor, histone subunit3, arginine kinase, HSP70 and HSP90 proteins using its template structure. The insect proteins with a QMEAN score were established for the development of models and for final confirmation, the protein model with a below -4 score was chosen.

The molecular docking analysis was performed using SAMSON software 2020 (https://www.samson-connect.net/) to study the interaction between the target proteins of *L. trifolii* and the ligand structures of BSTC compounds, to determine the direct and indirect effect of the BSTC compounds on insects survival. The sequence of each protein was downloaded from NCBI (https://www.ncbi.nlm.nih.gov/) in FASTA format to build the 3D structure binding models using TASSER server (https://zhanglab.ccmb.med.umich.edu/I-TASSER/). The affinity minimization was performed using 3DREFINE server (http://sysbio.rnet.missouri.edu/3Drefine/index.html). Pre-docking, all water molecules and ligands were removed while hydrogen atoms were added to the target proteins. On the other hand, the ligands were downloaded from PubChem (https://pubchem.ncbi.nlm.nih.gov/) in SDF format, and then converted into MOL2 format by using openbable software (http://openbabel.org/wiki/Main_Page). Interaction of *L. trifolii* proteins built models with the ligand structures of the common bean plant and the BSTC. Docking of the proteins was performed against the tested compounds by the aid of SAMSON 2020 software. The calculations of binding free energies were done using scoring function of AutoDock Vina as element in its script. Followed by the exhaustive search, 100 poses were analyzed and the best scoring poses were chosen to calculate the binding affinity of the ligands.

## Supplementary Information

Supplementary Information.
