# Supplementary material for: Assessing the effects of a novel biostimulant to enhance leafminer resistance and plant growth on common bean
Source: Sci Rep. 2021 Oct 8;11:20020. doi: 10.1038/s41598-021-98902-z (PMC8501134; doi:10.1038/s41598-021-98902-z)
Supplement: Supplementary file 1 — Supplementary Information. [file 41598_2021_98902_MOESM1_ESM.pdf]

Table S1: Primer pairs used for quantitative real-time polymerase chain reaction (RT-qPCR) analysis to characterize gene expression used for quantitative real-time polymerase analysis.

Group	Gene	Description	Sequences
Pathogenic related	PvPR1	Pathogenesis-related PR-1	F: AAAGCCAAGAGCGATTCTCTTTTCA<br>R: GAACACTCTGATTTGATAACACTTC
	PvPR3	Chitinase class I	F: ATTGTTGTGCCAATCCCTTT<br>R: CACCGCCATACAGTTCAAAA
Oxidative stress	SOD	Superoxide dismutase Access No. KF033693.1	F: GTGACATTCTTCCGGCTTTC<br>R: GCCCAGCCAGAACCGAATTG
	GST	Glutathione *S*-transferase	F: AGCTCTTCAAGGACACTGAGCCAA<br>R: AAAGGCTGTGGATGCTGCACTAGA
	PvPOD	Peroxidase	F: TCCTTTTCAGCACTTTCACT<br>R: AGAAAGCAGTGTTCTTGTGG
Defense and stress related pathway	PvHPRP	Hypersensitive-induced response protein	F: ATTGCATGGTTCATAGCCAGT<br>R: CCTCCACACAAGTATCAAAGGA
	PvPOX	α dioxigenase	F: CACAAATCCTCCCAAAATGG<br>R: AAAGGTTCACACCATTGATTGG
	Pvcallose	Callose synthase-like protein	F: TGGCTTAGTATTCGGATGTACCA<br>R: CGTTGTAATGAAAGCGAAGTGT
Phenyl propanoid pathway	PvPAL	Phenylalanine ammonia-lyase	F: GACACACAAGTTGAAGCACCA<br>R: TGCAGCTTCTTAGCATCCTTC
	Pv4CL	4-coumarate CoA-ligase	F: AGGTTGTTGGTGCTGAGAATG<br>R: CCAACCAAGTCAAAGATTCCA
Light-harvesting chlorophyll Type I	Lhcll-I	Chlorophyll a/b-binding protein Access. No. KF033510.1	F: ACCCTTCTCTCCACTTTCACT<br>R: ATGGTGCCTCACCGGAAAAT
Housekeeping	Act11	Actin11	F: TGCATACGTTGGTGATGAGG<br>R: AGCCTTGGGGTTAAGAGGAG

Table S2: Comparison of Growth performance of foliar fertilized *P. vulgaris* cvs Hama and Mualid with BSTC with sprayed with water (C1), yeast extract (C2) as controls. The percentages were calculated for the means of the seasons 2019 and 2020.

AlHama<br>increase % compared to BSTC treated									
**Item**	**Total yield (g)**	**Yield/plant (g)**	**No. of pods**	**Pod weight (g)**	**Plant height (cm)**	**Leaves no**	**TSS**	**Thickness**	**Chlo**
C1	150%	39%	50%	30%	54%	69%	33%	14%	15%
C2	70%	0.50%	27%	20%	28%	22%	20%	14%	10%
**Moraleda<br>increase % compared to BSTC treated**									
**Item**	**Total yield (g)**	**Yield/plant (g)**	**No.of pods**	**Pod weight (g)**	**Plant height (cm)**	**Leaves no**	**TSS**	**Thickness**	**Chlo**
C1	96%	16%	23.50%	39%	22%	73%	25%	59%	13%
C2	11%	6%	14.50%	19%	17%	40%	10%	35%	7%

Table S3. Optimized dock scores of biobased stimulator (BSTC) compound against *Liriomza trifolii* insect proteins.

Protein		Discovered compound		Score (ID) Kcal/mol
**Name**	**Accession Number (UniProt)**	**Pchem (ID)**	**Ligand**	
**Acetylcolenestrae**<br>X : 27.628<br>Y : 81.957<br>Z: 1.302	**Q5DV56**	**135414245**<br>135565649<br>13730	**Disodium 5'-inosinate**<br>Calcium 5'-guanylate<br>2D-Deoxyadenosine	**-5.5**<br>-5.4<br>5.0
**Elongation factor**<br>X: 134.00<br>Y:346.17<br>Z: 9414	**A0A240FEK0**	**443650**<br>5281643	**Delphinidin 3-glucoside**<br>Hyperoside	**-9.2**<br>-9.1
**Histone subunit3**<br>X: 68.974<br>Y: 35.535<br>Z: 15.172	**A0A240FEK2**	5281643	**Hyperoside**	**-7.0**
**Argenin kinase**<br>X:16.864<br>Y: 4.614<br>Z: 1.907	**A0A240FEK5**	**5281643**<br>443650<br>5280961	**Hyperoside**<br>Delphinidin 3-glucoside<br>Genistein	**-8.8**<br>-8.8<br>-8.3
**HSP70**<br>X: 1.459<br>Y: 13.128<br>Z: 3.936	**A0A240FEJ7**	**135414246**<br>135398635<br>135398641<br>443650<br>135565649	**Disodium 5'-guanylate**<br>Guanosine<br>Inosine<br>Delphinidin 3-glucoside<br>Calcium 5'-guanylate	**-9.0**<br>-8.8<br>-8.7<br>-8.6<br>-8.5
**HSP90**<br>X: 15.273<br>Y: 24.814<br>Z: 13.380	**A0A240FEJ6**	**443650**<br>5280961<br>5281643<br>443650	**Delphinidin 3-glucoside**<br>Genistein<br>Hyperoside<br>Delphinidin 3-glucoside	**-10.2**<br>-9.8<br>-9.8<br>-8.3

Figure S1

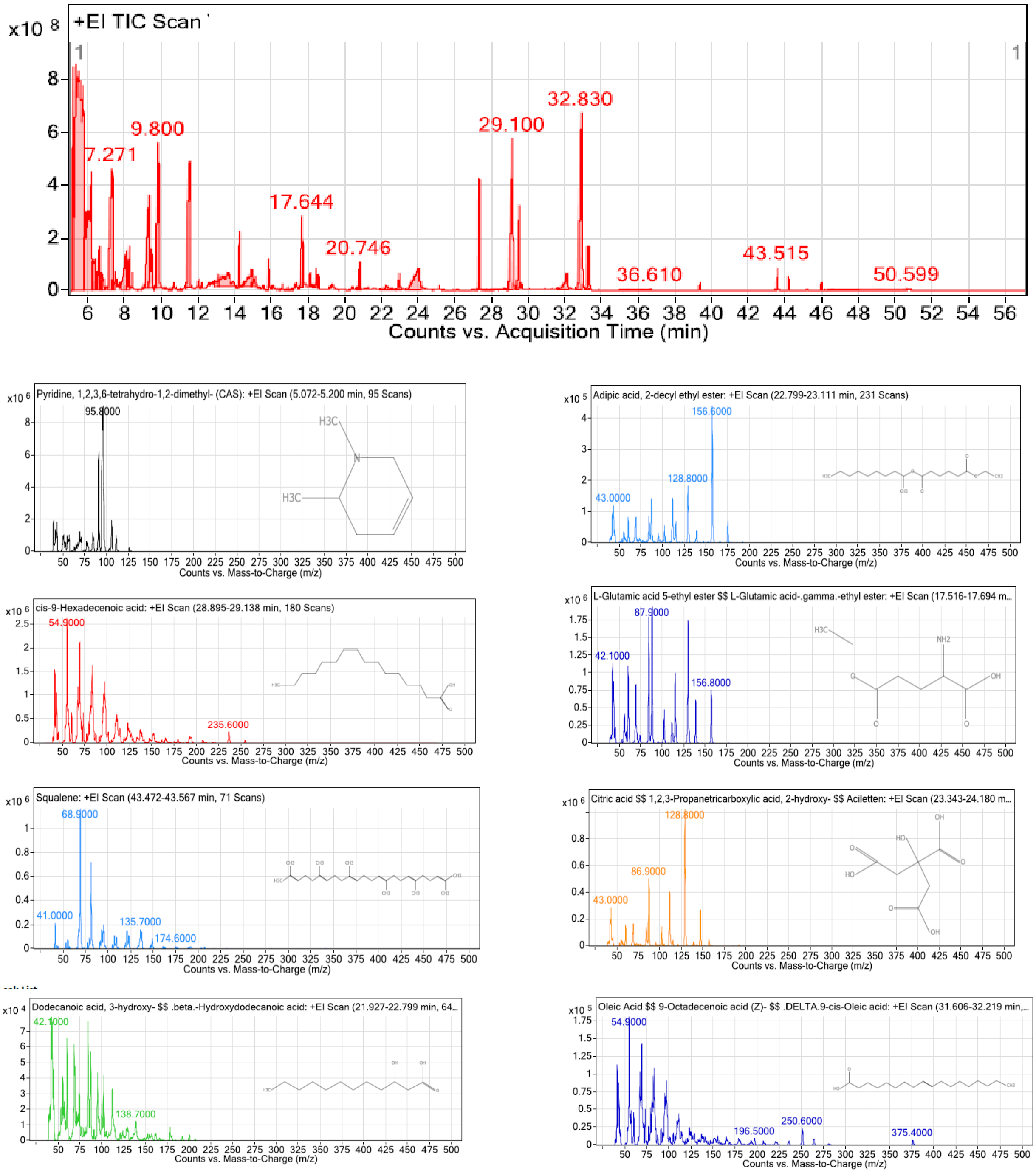

GC-MS GC-MS spectra for BSTC highlighting on pyridine 1,2,3,6 tetrahydro-1, 2-dimethyl, adipic acid ethyl ester, cis-9-Hexadecenoic acid, L- Glutamic acid 5-ethyl ester, squalene, citric acid, dodecanoic acid,3-hydroxy and oleic acid as important chemical composition that may have essential role induce immunity and resistance against many pathogens and insects.

Figure S2

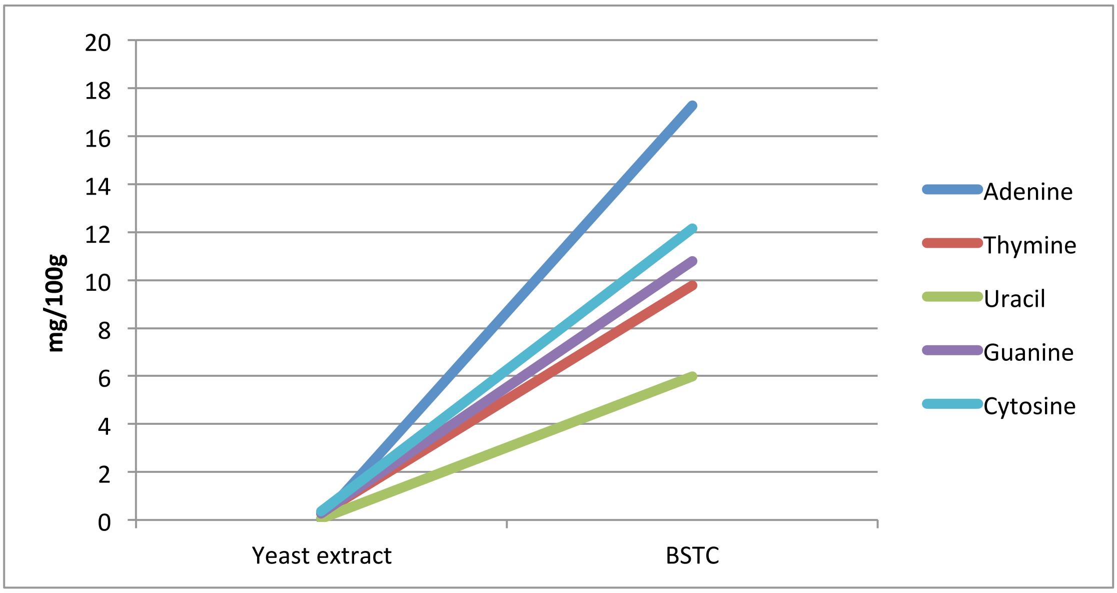

HPLC results for nucleobases concentration released from yeast RNA and DNA during BSTC preparation.
